# Mapping Emission Directionality of Color Centers in Silicon Carbide Microparticles

**DOI:** 10.1021/acsnanoscienceau.5c00187

**Published:** 2026-04-14

**Authors:** Erlend Lemva Ousdal, Marianne Etzelmüller Bathen, Anna Stray Rongve, Benedicte Allum Pedersen, Gard Momrak Selnesaunet, Snorre Braathen Kjeldby, Anne Marie Moen, Andrej Kuznetsov, Lasse Vines

**Affiliations:** † Department of Physics/Centre for Materials Science and Nanotechnology, 6305 University of Oslo, 0316 Oslo, Norway; ‡ Washington Mills AS, NO-7300 Orkanger, Norway

**Keywords:** Photonics, Silicon Carbide, Quantum Technology, Microparticles, Cathodoluminescence, FDTD, Luminescence Directionality

## Abstract

Color centers in semiconductors constitute a promising platform for developing quantum technology applications. To fully mature the platform, strict requirements on controlling the directionality of photon emission must be met. This work presents a method for studying and evaluating geometric features that can impact the directionality of emission from color centers in semiconductors by leveraging different nanostructures in silicon carbide (SiC) microparticles. Using spectrally resolved and angle-resolved cathodoluminescence measurements, we demonstrate decoupling of the color center emission from other luminescence sources and investigate how geometric features affect emission directionality. Combined with finite difference time domain simulations, geometric effects on emission directionality in the SiC particles are explored using simulations of luminescence from idealized geometries. As such, we present a holistic approach to evaluate the directionality of emission from color centers, independent of the material system, by combining experimental measurements and numerical simulations, using SiC as a benchmark case.

## Graphical abstract

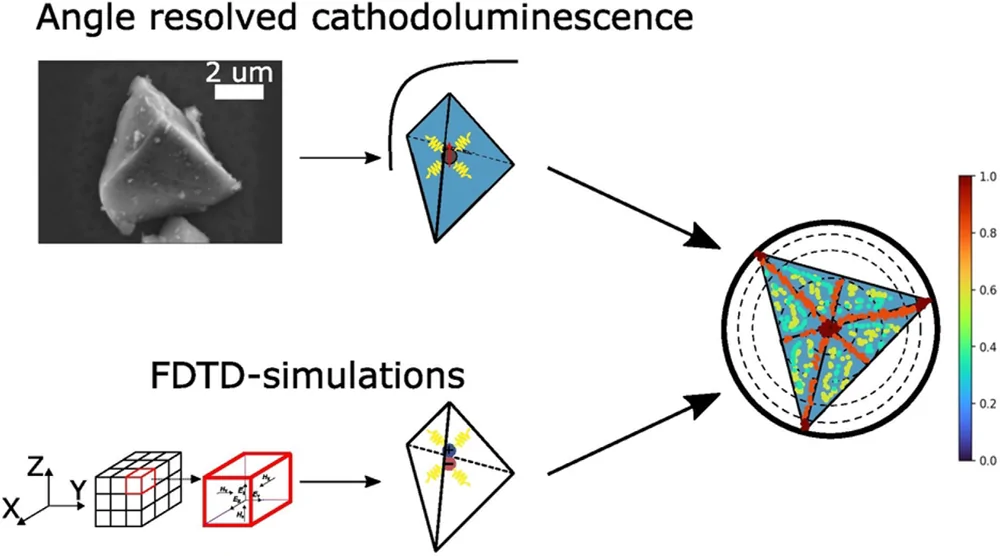

In the quantum technology (QT) platform based on color centers in semiconductors, photons serve as the read-out interface of spin-qubit systems as well as potential quantum information carriers in quantum computation and communication.^1–4^ Thus, adequate control of the color center emission directionality through, for example, structuring or waveguiding coupled to the readout circuit is an important strategy to achieve devices with high quantum efficiency.^5–7^ Generally speaking, guiding photons in a desired direction is possible using several approaches, including engineering of geometric shapes, antireflective interfaces, cavities, or exploiting subwavelength dimensionality for mode-confinement.^5,8–14^

Silicon carbide (SiC) is a promising QT material because it combines advanced nanofabrication capabilities with a plethora of color centers. Therefore, color centers in SiC acting as single-photon emitters (SPEs) have been studied extensively in recent years, revealing their potential for scalable quantum devices operating at room-temperature.^15–18^ In particular, the silicon monovacancy or V_Si_ has been investigated in detail. This defect has been found in various SiC polytypes, including 6H,^19,20^ 4H,^19–21^ 3C^22^ and 15R.^23^ Depending on the polytype, V_Si_ can occupy different hexagonal (*h*) and pseudocubic (*k*) lattice sites, thereby emitting at different wavelengths.^2,19,20,24^

In this work, we use V_Si_ as a test case to study the effects of geometries on the emission directionality. To study such effects on V_Si_ emission, we used a SiC powder, provided by Washington Mills, consisting of particles of randomly distributed geometries and sizes. This enabled the evaluation of how geometric features impact the emission efficiency, and how the mode confinement was a function of the particle size. In addition to the geometric variations, the particles consisted of four different SiC polytypes, enabling monitoring of polytype-dependent variations for equivalent defects. By combining spectrally and spatially resolved cathodoluminescence (CL) measurements of V_Si_ signatures with finite difference time domain (FDTD) simulations of emissions from particles with different geometries, we propose an approach that can be used to study the effects of emission directionality in naturally occurring and/or engineered structures. Thus, our data paves the way for designing and manufacturing structures that can enhance emission from color centers, essentially independent of the material system.

A scanning electron microscopy (SEM) image of the powder distributed on a Si-substrate, having an average size of 2 μm-5 μm (as measured by the provider, Washington Mills), is shown in Figure 1. The powder consists of 80% 6H-SiC, while the remaining 20% contains a mixture of the 4H, 3C and 15R SiC polytypes.^25^ Further details about the powder and sample preparation are provided in the SI. The powder was implanted with 1.8 MeV protons to generate V_Si_ in the irradiation tail, as calculated from SRIM simulations (Stopping and Range of Ions in Matter^26^) and shown in the SI. The threshold displacement energies for C and Si used in the SRIM simulations were 20 and 30 eV, respectively, which are values commonly employed in the literature.^27^ The resulting vacancy concentration is approximately 3 × 16 cm^–3^ considering 97% dynamic annealing.^27^ SRIM simulations also predict that vacancies are generated evenly throughout the particle for sizes up to ∼10 μm. Thus, the distribution of luminescent defects is assumed to be uniform in all particles studied herein.

**Figure 1:**
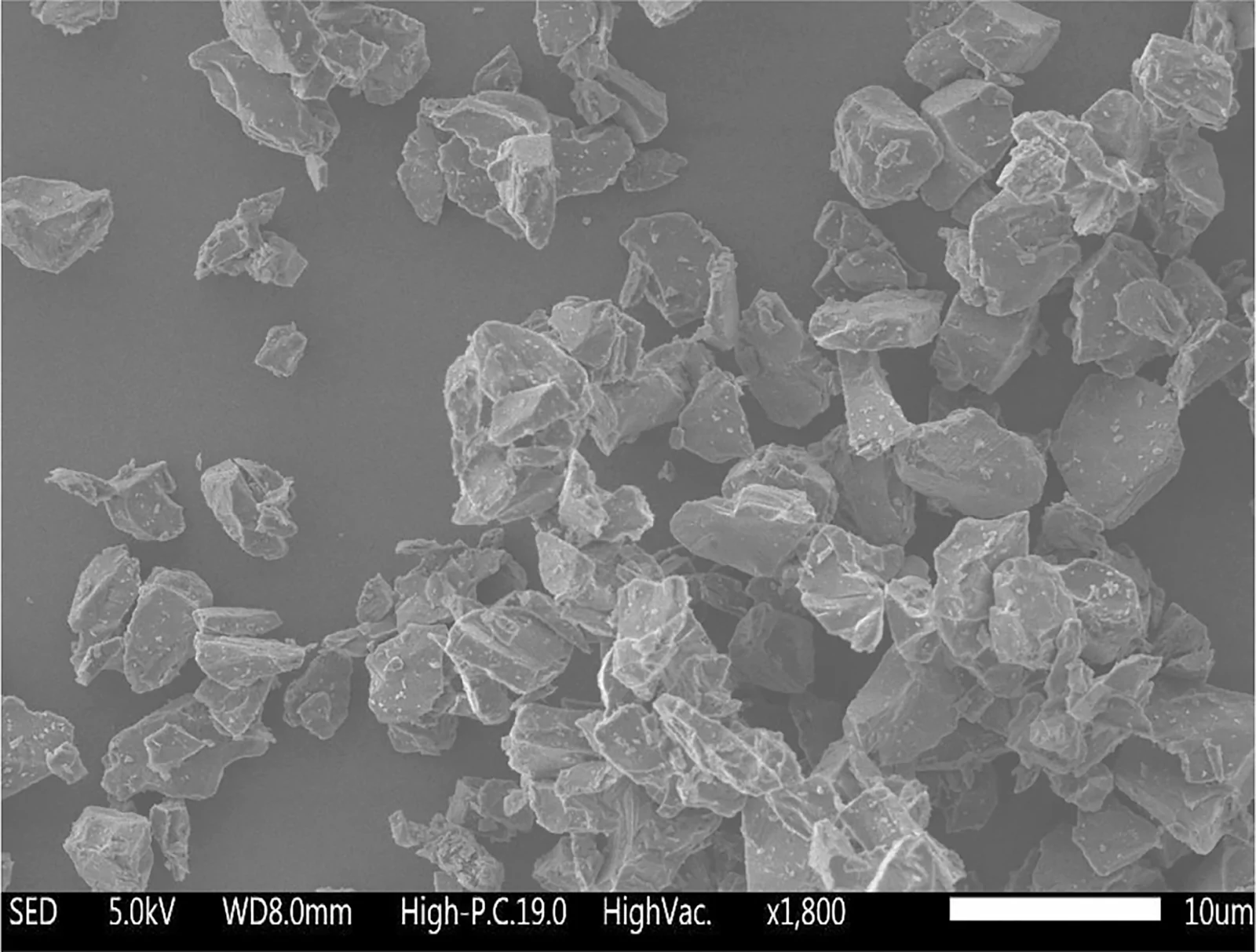
Scanning electron microscopy image of SiC microparticles. The particles are used as differently shaped/sized objects in this study. Particles are bonded to a Si-substrate with van der-Waals forces.

To measure color center luminescence from SiC microparticles as shown in Figure 1, we used CL and angle-resolved cathodoluminescence (ARCL) as implemented in the SEM and with narrow band-pass filters. The band-pass filters had a full width half-maximum bandwidth of 10 nm, allowing us to monitor emission related to the V_Si_ only. The central wavelengths of band-pass filters were selected depending on the polytype to isolate the relevant emission, i.e., at 860 nm for the following zero phonon lines (ZPLs): V1 (4H, 6H and 15R), V2 (15R), and at 920 nm for V2 (4H and 6H). Table 1 contains an overview of known ZPL wavelengths for emission from the V_Si_ in different polytypes of SiC. Further, when analyzing the luminescence data, a normalization protocol is introduced to account for differences in brightness resulting from factors not related to the vacancy emission itself, such as the height and angle of a particle surface. Normalization is done by dividing the whole CL-spectrum by the emission at 830 nm, a wavelength where there is a minimal number of contributions to the luminescence counts.^28,29^ The normalization allows the comparison of measurements between particles and also measurements at different points on a single particle, as shown by the example in the SI.

**Table 1:** Emission Wavelength of Luminescence from V_Si_ in Different Polytypes of SiC

Polytype (lattice site)	Labeling	Emission wavelength (nm)
4H (*h*)	V1, V1’	861, 858 ^19–21^
4H (*k*)	V2	915 ^19–21^
6H (*h*)	V1	861 ^19,20^
6H (*k*_1_)	V2	880 ^19,20^
6H (*k*_2_)	V3	920 ^19,20^
3C (*k*)	E-line	648 ^22^
15R (*k*_1_)	V1	862 ^23^
15R (*k*_2_)	V3	903 ^23^
15R (*k*_3_)	unknown/V2	860 ^23^

To support the interpretation of the CL data and predict effects on emission directionality, FDTD (finite difference time domain) simulations of Si vacancy emission from different geometric structures were performed in Tidy3D.^30–32^ Figure 1 demonstrates that the SiC powder encompasses a wide range of particle shapes and sizes. To reproduce this variability, we simulated objects with different geometries (sphere, cube, cone, triangle, or chevron) to enable comparison with experimental data. Moreover, we varied the sizes of all geometric objects so that the critical size, *L*, of the object was either half, equal or double of the emission wavelength used in simulations, specifically 860 nm corresponding to the V1 ZPL in 6H- and 4H-SiC.^19,20^ The critical size, *L*, describes the dimension of the key feature studied in each object, varying with the feature of interest to reveal its effect on the system. For the sphere, *L* equals the radius, while for the cube, *L* equals the side lengths. For the cone, *L* is equal to the radius of the base of the cone, while for both the triangle and chevron, *L* is equal to the thickness of the object. Additional details on the simulation environment are presented in the SI. Some examples of previous work that have combined experimental measurements and simulations to study luminescence directionality include the study of microparticle emission by combining luminescence measurements and optical modeling^33^ and semiconductor nanoparticle layers using luminescence measurements and full-wave finite-element simulations.^34^ The approach presented herein adds to this by enabling the evaluation of randomly occurring geometric features found in powder particles, while allowing simulations of arbitrary geometries to be experimentally verified through comparison with corresponding particle morphologies. As such, the effect of geometric features on emission directionality can be studied without the need for advanced nanofabrication.

To study the impact of geometrical factors on the color center emission experimentally, particles with different geometries were chosen for CL investigation. Figure 2 (left panel) shows the CL emission spectra from particles exhibiting variations of triangular features, as shown by the SEM images on the right-hand side. As shown by the gray dashed vertical lines, Figure 2 (left panel) reveals groups of signatures consistent with V_Si_ in the 6H-SiC polytype labeled as V1, V2 and V3. Importantly, since there should be no difference in the color center concentration between the particles as discussed above, the observed emission variation between particles in Figure 2 is likely due to variations in size and geometry of the particles. Notably, the emission may also be affected by strain as previously observed in ref.,^25^ which may be responsible for the spectral shift of the emission between particles in Figure 2.

**Figure 2:**
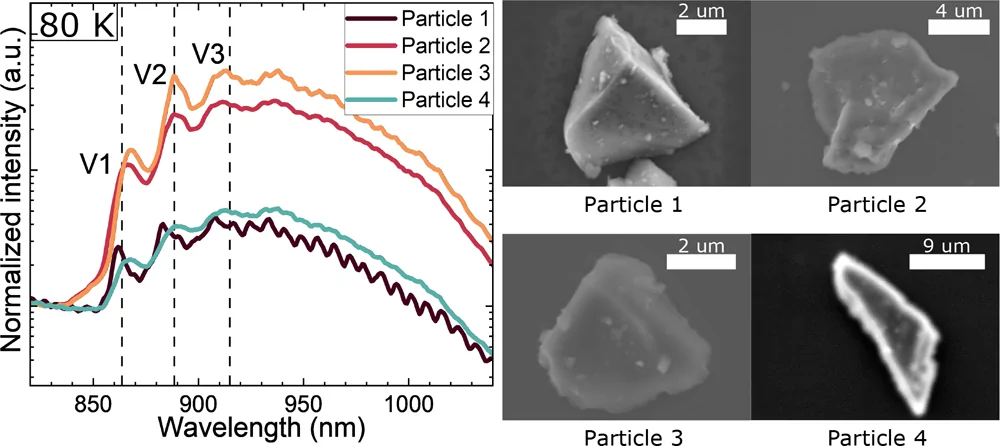
Emission variations in triangle shaped particles of the same polytype. The leftmost panel shows CL emission spectra of four 6H-SiC particles collected using a current of 1 nA and an acceleration voltage of 10 kV at 80 K temperature. The data is normalized to emission at 830 nm for each spectrum. As illustrated in the rightmost panel by SEM micrographs giving detailed information on the shape and size of the particles. Emission was collected from an average of multiple excitation spots over the whole particle, with a spot size of approximately 1 μm^2^.

Figure 3(a) and (b) clearly demonstrates emission variations across a particle of oblong shape, comparing data from different positions (positions 1–6) of the 6H-SiC particle surface. Specifically, CL-spectra from positions 1–3 and 5 exhibit three prominent emission lines, labeled V1–V3, corresponding to the different V_Si_ configurations in 6H-SiC. The emission intensity of the V-lines is shown to vary substantially depending on the position being probed on the microparticle. Importantly, in position 5, the three signatures are shifted with respect to what is expected for V-line positions in 6H-SiC, likely due to strain-induced shifting of the ZPLs.^25^ Moreover, in positions 4 and 6, a broad emission spectrum in the range corresponding to that of V_Si_ is clearly visible (Figure 3a), even though the V_Si_-related ZPLs are quenched. Interestingly, although positions 5 and 6 are right next to each other, see Figure 3(b), vacancy related emission is brighter in position 6 than in position 5.

**Figure 3:**
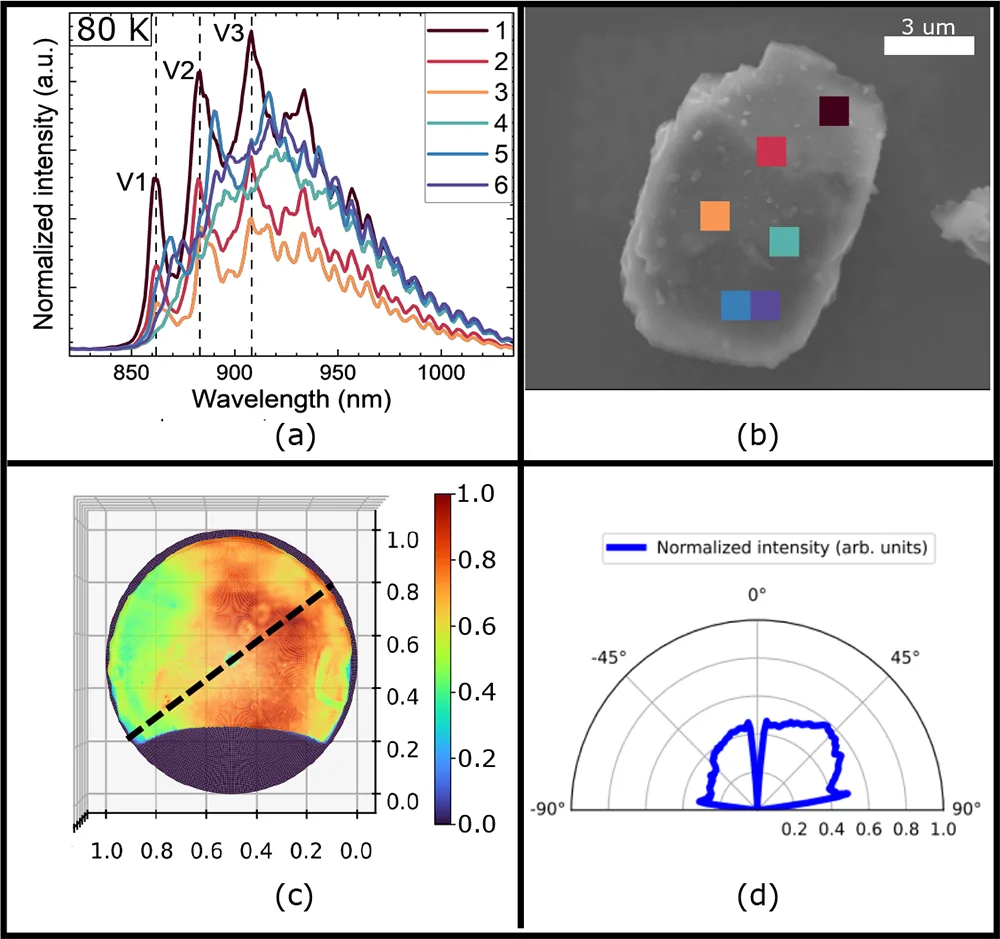
Emission variations across an oblong 6H-SiC particle. (a) CL spectra collected from different positions as traced in panel (b). Panels (c) and (d) show an ARCL heatmap and cross-section polar plot, respectively, of the same particle. The cross-sectional polar plot is taken at the diagonal of the particle as illustrated by the dashed line in panel (c). The data in this figure is collected without use of a band-pass filter.

We attribute the variation in V_Si_-related emission intensities in the oblong particle, shown in Figure 3(b), to geometrical features formed during particle fabrication. To verify this, we employ ARCL measurements to visualize how the emission from a single excitation spot depends on the particle geometry. Figures 3(c) and (d), via an ARCL heatmap and a cross-section polar plot, respectively, demonstrate that luminescence is not uniformly emitted from the particle. The heatmap in Figure 3(c) shows the normalized angular dependence of emission, demonstrating that the emission is more intense (red) toward the top and rightmost parts of the particle. In comparison, emission from other regions is sparse, especially toward the left side of the particle. The cross-sectional polar plot in Figure 3(d) is taken along the dashed line in Figure 3(c), and helps to evaluate the emission distribution in the *xy*-plane. Figure 3(d) shows that the emission from the particle does not have a Lambertian profile, as the emission is strongest at 45°, from the top right side of the particle, while emission from the left side of the particle is mainly directed toward 0°. It should be noted that even though the measurement picks up luminescence from the whole particle, the primary beam is kept constant at the center of the particle. As such, the ARCL emission pattern contains information about the ”takeoff” angle of the emission from all parts of the particle, but detailed analysis and comparison with simulation is required to disentangle the contributions of different geometries of the particle.

Figure 3(c) and (d) shows all SiC emission in the 650–1050 nm spectral range and do not distinguish between emission from V_Si_ and other sources. To isolate the emission from V_Si_ during ARCL measurements, narrow band-pass filters with central wavelengths of 860 and 920 nm are employed. Figure 4 compares the emission of the particles in the 650 nm–1050 nm spectral range with that of the narrow emission bands centered around the V1 and V3 ZPLs associated with V_Si_ in 6H-SiC for five particles of different shapes. As previously shown for broadband emission in Figure 3(c) and (d), the particles all have a preferred direction of emission, meaning that the emission is not uniform in all directions. For all particles in Figure 4, the excitation beam-spot was directed at the center of the surface of each particle. Further, the emission depends strongly on the particle shape, with the preferred direction following the particle morphology. In particular, sharp edges promote higher emission intensity than flat or smooth surfaces. However, not all sharp edges have this effect which is attributed to the specific dimensions of the particle features and how they couple with the emission wavelength. Interestingly, the filtered narrow-band emission at around 860 and 920 nm has a higher directionality than the broad-band (nonfiltered) measurements. This is attributed to the fact that the emission at these wavelengths couple differently with the geometric features than emission from other sources. One interpretation of the data shown in Figure 4 is that the emission from sources other than V_Si_ is more uniformly distributed. If this is the case, the contrast in the nonfiltered ARCL spectra will be mainly due to V_Si_, which could explain why the direction of emission is largely the same with and without filtering.

**Figure 4:**
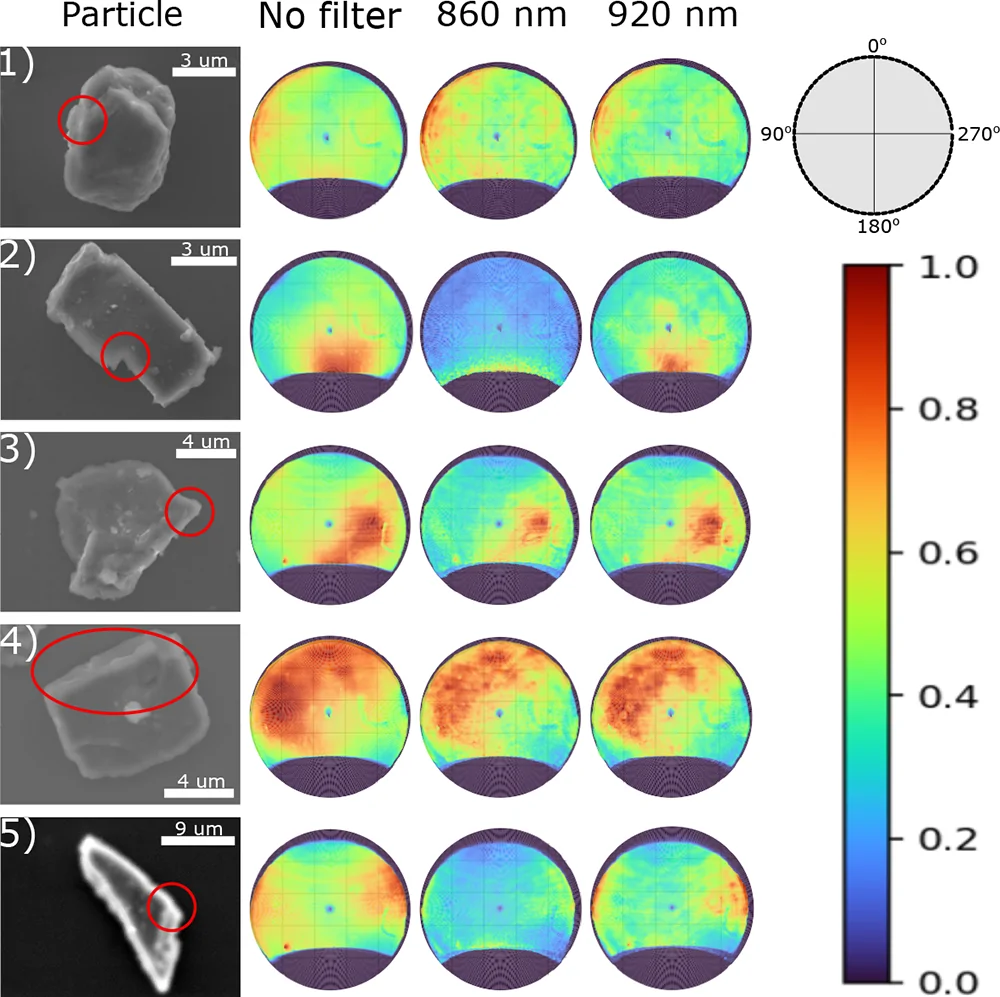
Emission directionality from various SiC particles. The first column shows SEM images of five SiC microparticles: obling, rectangular, feature-rich, square and triangular. The three next columns show ARCL heatmaps of the five different SiC particles with no filter, filtered at 860 nm and filtered at 920 nm, in order from left to right. All ARCL-figures are normalized to the brightest emission in each measurement, as to minimize the effect of acquisition time and differences in brightness between each particle.

We will now consider the emission from each of the five SiC particles shown in Figure 4 sequentially, with directions described according the coordinate system in the figure. For the first particle, of oblong shape with varied morphological features on the surface, emission is mainly directed toward 90°, from the region marked with a circle, in the ARCL-heatmap. Employing optical filters at 860 and 920 nm results in enhanced emission variations due to the geometric features of the particle, as it has multiple edges and corners. The second particle, of near-rectangular shape, emits strongly toward 180° in Figure 4. The emission from V1 and V3 is rather different in this particle, as V1 is only directed toward 180°, while V3 is also emitting in other directions though most of the emission from V3 is directed toward 180° as well. This particle has a flatter surface than the first particle, as shown in the leftmost column of Figure 4, and the enhanced emission is formed in the direction of the chevron at the bottom side of the image, marked with a circle, suggesting that this feature promotes an enhancement of emission. Particle-3 clearly exhibits enhanced emission between 180°–270°. Looking at the shape of the particle, the region with increased emission has a sharp edge in this direction, suggesting that this feature enhances emission. On the far right side of the particle, a sharp horn marked with a circle is observed. Looking at the filtered ARCL-spectrum, we see that the emission at both 860 and 920 nm is more centered around this horn than in the unfiltered spectrum, suggesting that this feature promotes emission from V_Si_. The fourth particle in Figure 4 has the shape of a rectangular cube, with enhanced emission mainly between 0°–90°, as shown by the ARCL heatmap. The brightest emission in the unfiltered case corresponds to what looks like a sharp edge in the SEM image, marked with a circle, suggesting that this enhances broadband emission. Applying the narrow filters at 860 and 920 nm shows that luminescence from the V1 and V3 ZPLs is emitted primarily in this direction as well. This could suggest that emission originating from other sources than V_Si_ is more uniform, and the angular contrast we measure without filters can be attributed to V_Si_-related emission, though this is not an unambiguous interpretation. Finally, particle-5 in Figure 4, shaped as a triangle, exhibits stronger emission perpendicular to the longest length of the particle. The strongest signal is directed toward 270°, which happens to be where we find a sharp angle in the particle, marked with a circle, supporting the idea that these features enhance the emission intensity of V_Si_ in SiC.

The above discussion shows that geometric features in the particles strongly influence emission directionality. This promotes the combination of SEM, CL and ARCL to gain insight into how emission from color centers is guided and redistributed by the particle geometry. To support and explain the experimental findings, we employ FDTD simulations of simplified microparticle geometries designed to reproduce the most prominent features observed in Figure 4. The geometries are chosen to study features that are similar to those observed in the real powder, allowing us to isolate and understand the contributions of individual morphological effects. It should be noted that the simulations consider an ideal dipole with perfectly coherent emission, while the emission from the real color centers has a finite coherence length. Nevertheless, we consider the spatial emission pattern, determined primarily by the interaction between the emitter and the surrounding geometry, which is not strongly affected by temporal coherence. Therefore, the luminescence simulations provide a physically meaningful comparison for analyzing geometric features in ARCL, even though the absolute interference contrast may differ.

The considered objects (Column-1) and resulting emission profiles for different object dimensions (Columns 2–4) are shown in Figure 5. Overall, the simulations confirm that both the shape and dimension of the SiC-object have a pronounced impact on the directionality of photons emitted from a point source embedded within the structure. Looking at a spherical geometry without sharp features or edges, as in Figure 5(a), we find, as expected, that the emission is uniformly distributed from the surface of the object. Experimental data confirm this, as we find little to no enhancement of emission directionality from round features (see, e.g., the rounded features of particle-1 and the top of particle-3 in Figure 4). Considering the critical dimension of the sphere, increasing in size from *L* = λ/2 to *L* = 2λ where λ = 860 nm represents the V1 ZPL of the V_Si_ in 4H- and 6H-SiC, results in emission being more confined to the center of the object (see Figure 5a). Comparing to the cube in Figure 5(b), we observe a similar impact on emission directionality for the cubic and spherical particle shapes when the side lengths are smaller than the emission wavelength, i.e., *L* = λ/2. This is expected since there is not enough space to generate even one mode of the given wavelength of 860 nm. However, in the case where the L = λ, a clear pattern appears, in which the emission is strongly directed toward the center and corners of the cube, indicating that such features have potential for directing emission. This also supports our experimental findings, where the most pronounced shape-dependent intensity enhancements were found for sharp edges (see Figure 4). Further increasing the cube size to *L* = 2λ (see Figure 5b Column-4), leads to a loss of emission through the cube corners. In this case, the majority of the emission is found directly in the center of the cubic geometry, which is incidentally also where the point source is situated.

**Figure 5:**
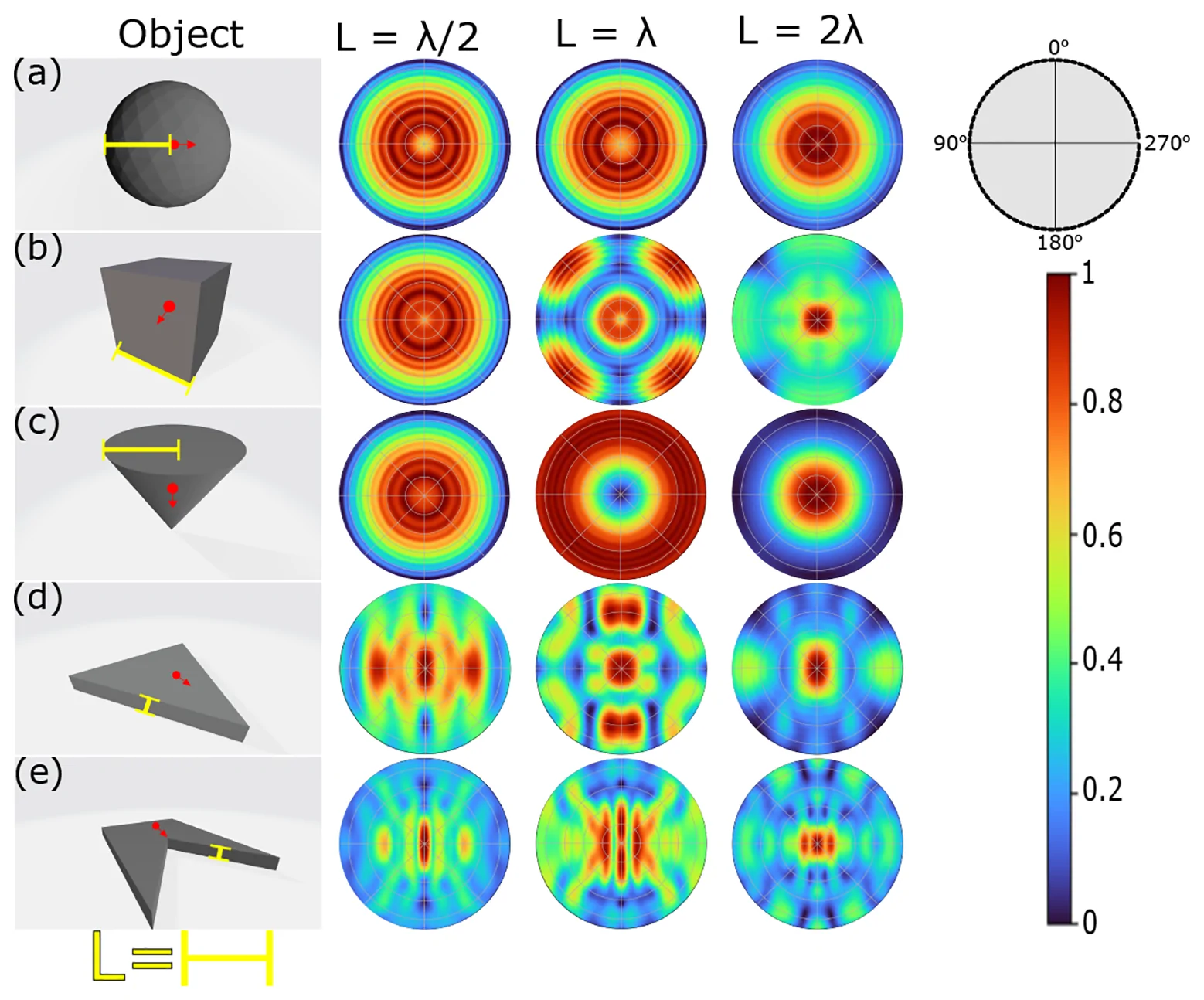
Simulated emission directionality from SiC particles. Normalized simulation results from Tidy3D simulations, using five different geometrical objects: (a) sphere, (b) cube, (c) cone, (d) triangle and (e) chevron. The objects are illustrated in Column-1. Each object has a critical dimension *L*, which is either half (Column-2), equal (Column-3) or double (Column-4) the emission wavelength from the V1 ZPL of V_Si_ in 4H- and 6H-SiC at λ∼860 nm. The source of emission (red circle) is positioned in the center of each object, as explained in detail in the SI, and data is collected in the direction of the red arrow.

Figure 5(c) shows the simulated emission profile from a color center contained within a SiC cone-structure. The monitor is positioned at the tip of the cone to investigate how a sharp tip affects emission directionality, and the critical dimension is the radius of the circle at the base of the cone. Clearly, the emission properties strongly depend on the size of the cone. If the diameter of the base is equal to the emitted wavelength (*L* = λ/2), the emission is evenly distributed in a similar manner to the sphere and cube shapes. This is most likely related to the fact that the structure is too small to contain any modes of the emission. Doubling the diameter (column marked by *L* = λ in Figure 5c) effectively removes any emission at the center of the cone, while most of the emission is distributed around the center. By doubling the diameter again, however, the opposite happens, and emission is strongly promoted in the direction of the tip of the cone (column marked by *L* = 2λ in Figure 5c). Again, simulations confirm that the sharp edges direct emission, as seen in particles 4 and 5 in Figure 4, further validating the comparison between ARCL measurements and FDTD simulations.

More complex shapes are found to cause strong variations in emission directionality. For example, Figure 5(d) shows the simulations of a slab with the geometry of a 3D-triangle, with the thickness being the critical size. The dimensions in the *xy*-plane are kept constant, with the side opposite to the tip at 4 μm and the other sides being of length 3.6 μm. By changing the thickness of the slab, the emission pattern changes dramatically. When the thickness is less than the wavelength of emission (column labeled *L* = λ/2 in Figure 5d), the emitted light is confined largely to the *xy*-plane, as wave modes can only form in this plane. As the thickness increases to be equal to the wavelength of emission (column labeled *L* = λ in Figure 5d), a larger portion of the emission is directed along the *z*-axis, as two intense spots appear above and below the center of the emission pattern. A further doubling of the thickness (column labeled *L* = 2λ in Figure 5d) leads to a centering of the emission, in the same way as we observe for the other geometries. This result is of interest due to how the *z*-component of emission changes as the triangular object thickness goes from below to equal that of the wavelength. The triangular shape largely resembles that of particle-5 in Figure 4. As particle-5 also shows enhanced emission toward the tips of the triangle, it shows that the same effect is observed in simulation and experiment, supporting the interpretation that emission intensity variations from the SiC microparticles are related to geometric and size variations. Additionally, simulation predicts that the thickness of the structures has a major impact on the emission pattern from color centers in SiC.

The last geometry that was simulated, shown in Figure 5(e), is that of a chevron—or the inverse shape to the triangular slab shown in Figure 5(d). This geometry resembles a very local environment in a rough surface, where multiple sharp features are separated by small distances. Comparable features are observed in, e.g., particles 2 and 3 in Figure 4. The simulated emission patterns of this object, as shown in the rightmost columns of Figure 5, demonstrate that photons are mostly emitted from the two ”horns” and from the center between them. Intriguingly, this is precisely what was observed for the edge in the second particle in the ARCL heatmaps of Figure 4, and for the horn-like structure in particle-3 in the same figure. Finally, and similarly to the other object geometries, as the thickness of the structure increases, a bigger portion of the emission comes from the center of the structure.

In this work, we measure the luminescence from V_Si_, and we compare the results with FDTD simulations that consider the directionality of emission from color centers embedded within nano- and microstructures of SiC. Overall, the most pronounced emission directionality is observed by ARCL from SiC particles with sharp edges. The supporting simulations provide the added restriction that the dimensionality surrounding the edge must be larger than the emission wavelength, which is indeed the case for the particles studied herein having average sizes of 2 μm–5 μm. As such, we find the combination of CL/ARCL with FDTD luminescence simulations to be a powerful tool for studying the effects of geometric features on emission directionality of color centers — here for the V_Si_ in SiC microparticles, but generally applicable to other materials and structures as well. This method can both help to understand and engineer features that promote emission directionality by employing geometries that are discovered to enhance luminescence, and to circumvent possible fabrication flaws by avoiding features that quench the luminescence. As such, the approach can contribute to maturing the color center platform for future advances in QT and discover novel approaches to control emission directionality.

## Supplementary Material



## Data Availability

The data that support the findings of this study are available from the corresponding author upon reasonable request.

## Notes

Experimental details of sample preparation, SRIM-simulation of H^+^-implantation, experimental details about cathodoluminescence setup, description of normalization method, and a description of the FDTD-simulation conditions (PDF)
