# Supplementary material for: Mapping Emission Directionality of Color Centers in Silicon Carbide Microparticles
Source: ACS Nanosci Au. 2026 Apr 14;6(4):523–9. doi: 10.1021/acsnanoscienceau.5c00187 (PMC13495454; doi:10.1021/acsnanoscienceau.5c00187)
Supplement: Supplementary file 1 [file ng5c00187_si_002.pdf]

Supporting information

Mapping emission directionality of color centers in silicon carbide microparticles

Erlend Lemva Ousdal,[1] Marianne Etzelmüller Bathen,[1] Anna Stray Rongve,[1] Benedicte Allum Pedersen,[1] Gard Momrak Selnesaunet,[1] Snorre Braathen Kjeldby,[1] Anne Marie Moen,[2] Andrej Kuznetsov,[1] and Lasse Vines[1]

[1)]*Department of Physics/ Centre for Materials Science and Nanotechnology, University of Oslo, 0316 Oslo, Norway*[a)]

[2)]*Washington Mills AS, NO-7300 Orkanger, Norway*

(Dated: 5 April 2026)

[a)]Electronic mail: erlendou@uio.no

I. SAMPLE PREPARATION

The silicon carbide (SiC)-powder used in this work is provided by Washington Mills, and is made by grinding bulk SiC into smaller particles having an average size of 2 µm–5 µm. The powder consists of 80 % 6H-SiC and the remaining 20 % consists of a mixture of the 4H, 3C and 15R SiC polytypes. Both size and polytype distributions are measured by Washington Mills. The emission wavelength from the silicon vacancy (V_{Si}) is dependent on the polytype, and relevant emission wavelengths are listed in Table I.

TABLE I: Emission wavelength of luminescence from V_{Si} in different polytypes of SiC.

Polytype (lattice site)	Labeling	Emission wavelength (nm)
4H (*h*)	V1, V1'	861, 858[1–3]
4H (*k*)	V2	915[1–3]
6H (*h*)	V1	861[1,3]
6H (k_1)	V2	880[1,3]
6H (k_2)	V3	920[1,3]
3C (*k*)	E-line	648[4]
15R (k_1)	V1	862[5]
15R (k_2)	V3	903[5]
15R (k_3)	unknown/V2	860[5]

To measure the SiC microparticles using optical characterization it is mounted on a Si-substrate by depositing powder on top of the substrate with a spatula, and then gently shaking the sample by hand. After shaking the samples, only the powder that adheres to the substrate via van der Waals forces remains.

The samples with the attached powder are then irradiated with 1.8 MeV protons to a fluence of 5×10^{14} cm^2 using a titanium hydride source to form Si vacancies. The projected range of 1.8 MeV protons is $\sim$30 µm in SiC, as simulated in Fig. 1 using the SRIM (Stopping and Range of Ions in Matter) code [Ziegler] and using displacement energies of 20 eV and 30 eV for C and Si, respectively[6]. Thus, only the defects formed in the irradiation tail of the protons remain in the SiC microparticles, with the Bragg peak of implantation falling in the Si substrate.

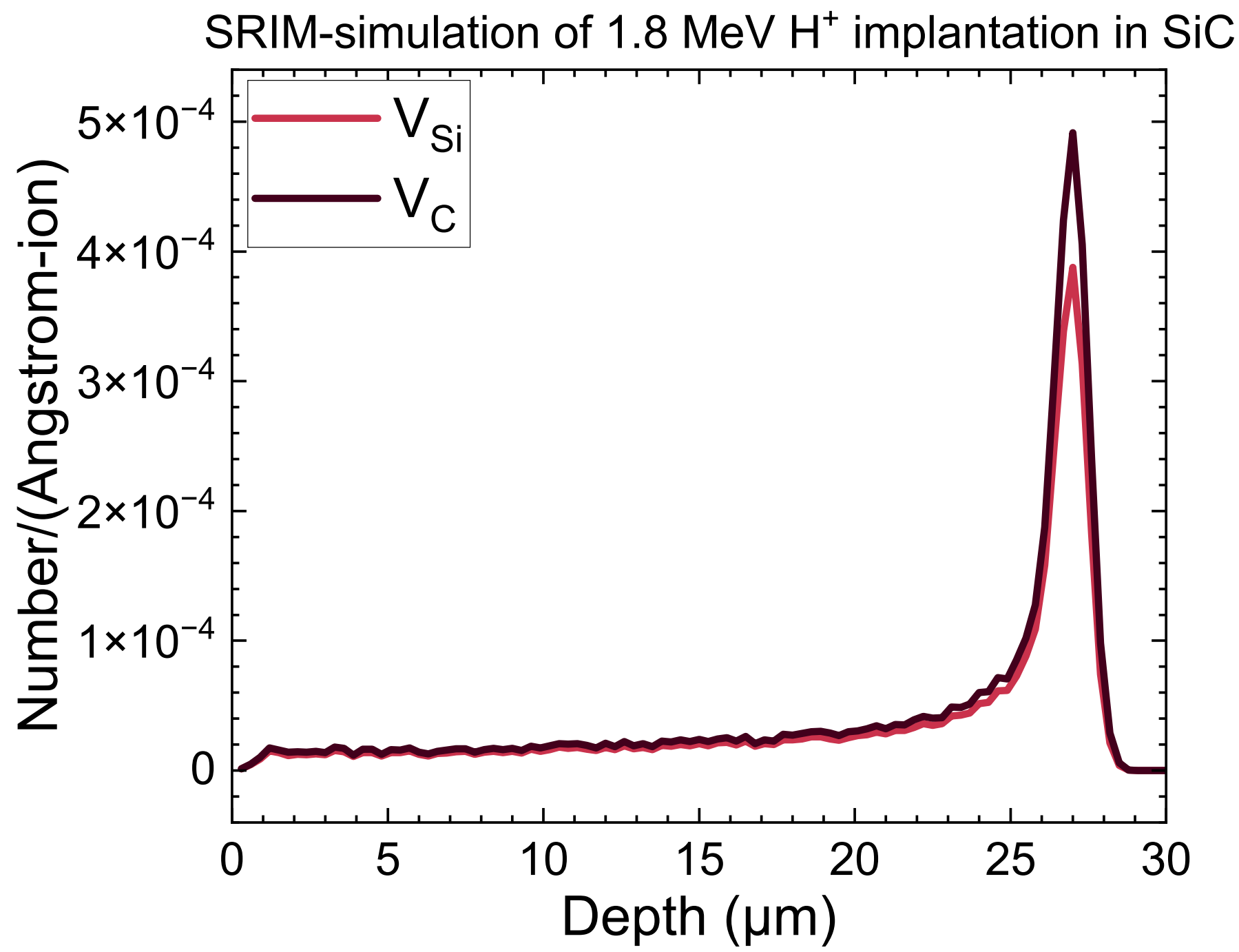

FIG. 1: SRIM simulation of 1.8 MeV H^+ in SiC, using a displacement energy of 20 and 30 eV for C and Si, respectively.

II. CATHODOLUMINESCENCE SETUP

The cathodoluminescence (CL) system that is used in this work is a SPARC-system provided by Delmic. All measurements are performed inside a JSM-IT300 InTouchScope Scanning Electron Microscope (SEM). The electron current used for the CL measurements is approximately 1 nA and the acceleration voltage is set to 10 kV. Simulations performed in Casino[7] (not shown) predict that primary electrons do not penetrate deeper than a standard particle size of 2 μm–5 μm, as the penetration depth in SiC at 10 kV is a maximum of 800 nm. Moreover, all measurements are performed at 80 K. The sample is cooled by flowing a cooled nitrogen gas through the sample stage. Cooling of the gas is achieved by passing it through a coil that is submerged in a liquid nitrogen bath.

To ensure that we can compare the intensity of V_{Si} between different particles, we normalize the CL to the emission at 830 nm, as this is a wavelength that has few contributions. Using this protocol, we make sure observed differences between particles are due to the emission of V_{Si} and not other factors such as surface effects. An example of the normalizing protocol is shown in

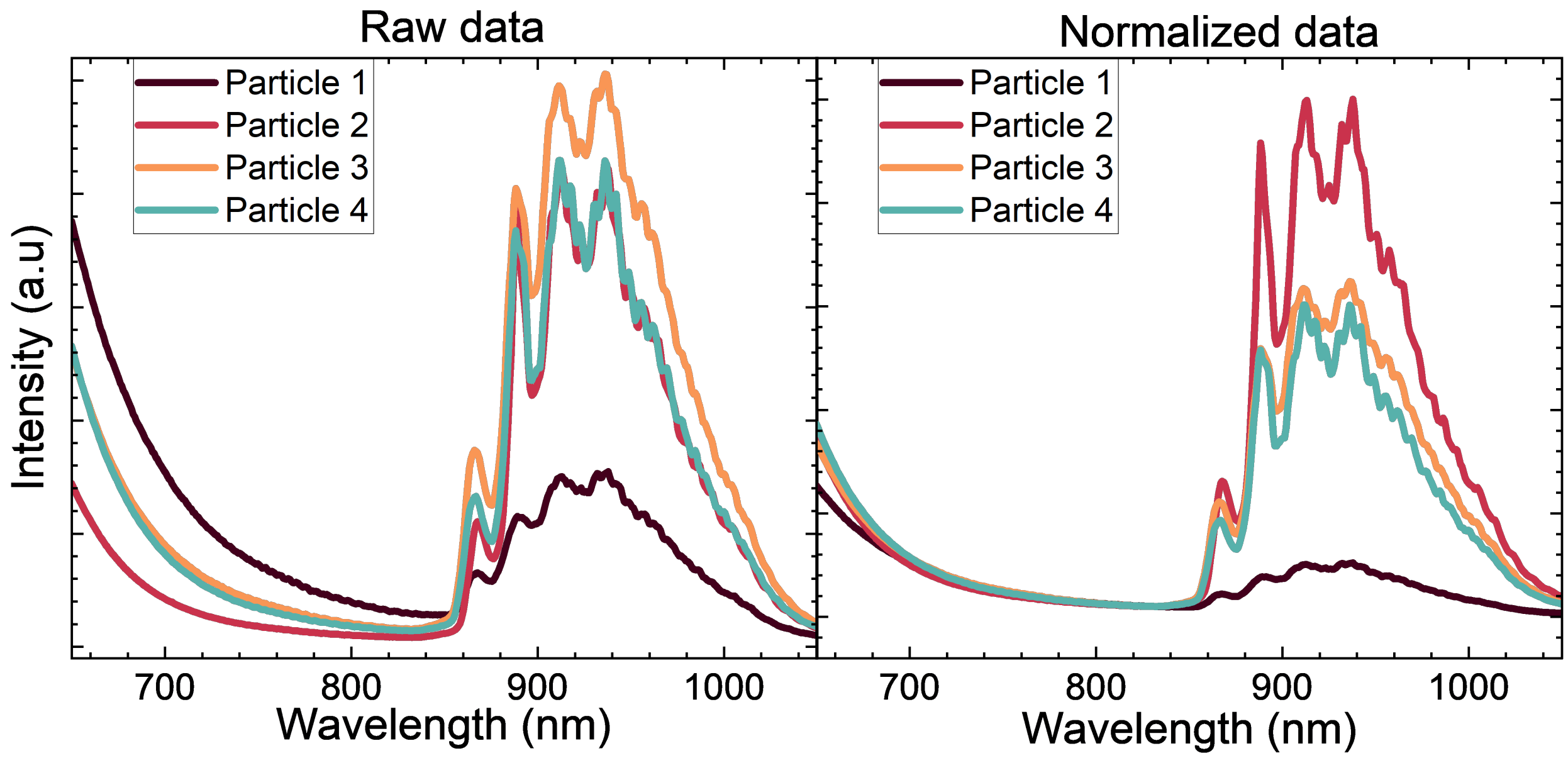

FIG. 2: Total emission (left) and normalized emission (right) of four different particles. The normalization protocol consist of dividing all emission raw data with the intensity of emission at 830 nm, as this is a wavelength with some luminescence, but with few contributions[8,9].

Figure 2

To measure the directionality of emission from point defects in the particles, angle resolved cathodoluminescence (ARCL) is performed. ARCL uses the same setup as regular CL, but the obtained information is the angular direction of the emission instead of spectral resolution. ARCL is measured under the same conditions as CL, though the dwell time is chosen as to get a maximum signal without saturating the detector. A schematic of the measurement setup is shown in Figure 3.

The CL setup can not obtain both spectral and angular information simultaneously. As a results, the collected ARCL signal contains all wavelengths that the grating can disperse, which is a range of approximately 400 nm. To enable probing of emission related to the V_{Si} alone, luminescence from the zero-phonon lines (ZPLs) of interest is filtered out. The two filters being used in this work have a central wavelength (CWL) of 860 nm and 920 nm, respectively. This corresponds to the wavelengths of the V1 and V3 ZPLs in 6H-SiC, the prime polytype being probed herein, and the V1 and V2 ZPLs in 4H-SiC. Both filters are produced by Thorlabs and have a full width at half maximum (FWHM) bandwidth of 10 nm.

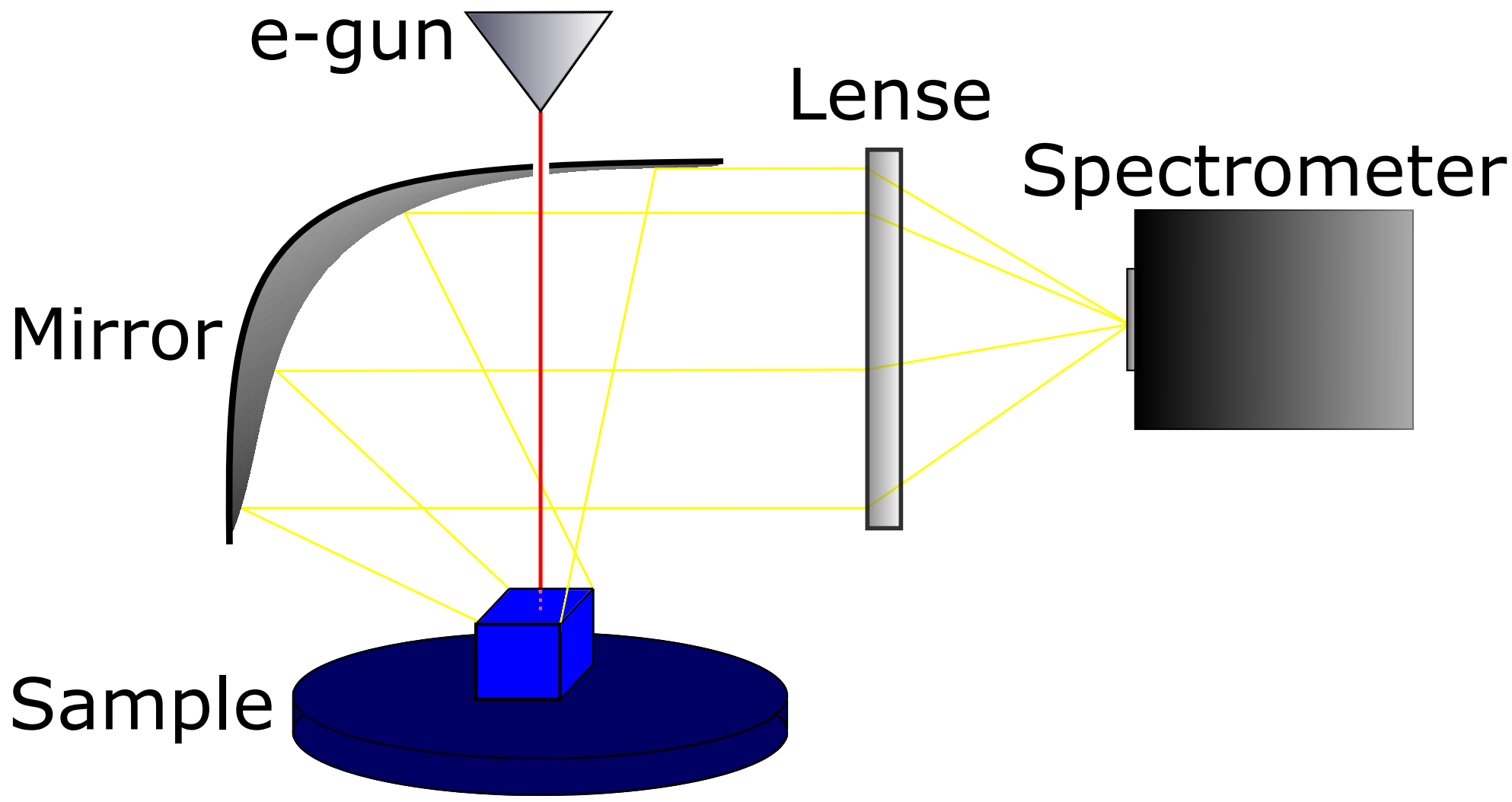

FIG. 3: Schematic representation of ARCL from a cube. Luminescence is emitted from the edges of the cube which are collected at different points on the mirror. The luminescence is further collected and processed in the spectrometer to give information about emission intensity with angular resolution.

III. SIMULATION CONDITIONS

Tidy3D, which is a GPU-based simulation tool used to perform finite difference time domain simulations of photonic systems[10–12], is used to simulate the propagation of luminescence from dipoles in objects with the refractive index dispersion of SiC. The shape of the objects is either a sphere, cube, cone, triangle, or chevron. The object is positioned in the center of the simulation environment, which is of size $20 \times 20 \times 20$ µm^3 and with perfectly-matched layer boundary conditions[11,12]. A polarized point dipole, emitting at 860 nm, is positioned in the center of the SiC-structure. To capture polarization-independent luminescence, three separate simulations were performed for each object, one for each dipole orthogonal polarization, after which the measured data was added together during post processing. A monitor is then positioned 9 µm from the defect dipole, spanning a 2D-area of 9×9 µm^2. The structures are then oriented such that sharp features are pointing towards the monitor, with the exception of the cube and the sphere. For the cube, the side of the cube is facing the monitor, while as the sphere is perfectly uniform, the monitor is simply placed at a distance from one edge of the sphere.

REFERENCES

REFERENCES

[1]E. Sörman, N. T. Son, W. M. Chen, O. Kordina, C. Hallin, and E. Janzén, "Silicon vacancy related defect in 4H and 6H SiC," Physical Review B **61**, 2613–2620 (2000).

[2]E. Janzén, A. Gali, P. Carlsson, A. Gällström, B. Magnusson, and N. Son, "The silicon vacancy in SiC," Physica B: Condensed Matter **404**, 4354–4358 (2009).

[3]M. Wagner, B. Magnusson, W. M. Chen, E. Janzén, E. Sörman, C. Hallin, and J. L. Lindström, "Electronic structure of the neutral silicon vacancy in 4H and 6H SiC," Phys. Rev. B **62**, 16555–16560 (2000).

[4]H. Itoh, M. Yoshikawa, I. Nashiyama, H. Okumura, S. Misawa, and S. Yoshida, "Photoluminescence of radiation induced defects in 3C-SiC epitaxially grown on Si," Journal of Applied Physics **77**, 837–842 (1995).

[5]T. Lingner, S. Greulich-Weber, and J.-M. Spaeth, "The ground state of silicon vacancies in 6H-SiC and 15R-SiC," Physica B: Condensed Matter **308-310**, 649–651 (2001), international Conference on Defects in Semiconductors.

[6]M. E. Bathen, A. Galeckas, J. Müting, H. M. Ayedh, U. Grossner, J. Coutinho, Y. K. Frodason, and L. Vines, "Electrical charge state identification and control for the silicon vacancy in 4H-SiC," npj Quantum Information **5**, 111 (2019).

[7]D. Drouin, A. R. Couture, D. Joly, X. Tastet, V. Aimez, and R. Gauvin, "Casino v2.42—a fast and easy-to-use modeling tool for scanning electron microscopy and microanalysis users," Scanning **29**, 92–101 (2007).

[8]A. O. Konstantinov, A. Henry, C. I. Harris, and E. Janzén, "Photoluminescence studies of porous silicon carbide," Applied Physics Letters **66**, 2250–2252 (1995).

[9]S. W. Lee, S. I. Vlaskina, V. I. Vlaskin, I. V. Zaharchenko, V. A. Gubanov, G. N. Mishinova, G. S. Svechnikov, V. E. Rodionov, and S. A. Podlasov, "Silicon carbide defects and luminescence centers in current heated 6H-SiC," Semiconductor Physics, Quantum Electronics & Optoelectronics **13**, 24–29 (2010).

[10]Z. Liu and J. K. S. Poon, "Comparison of Lumerical FDTD and Tidy3D for three-dimensional FDTD simulations of passive silicon photonic components". arXiv (physics.optics). 2025-06-20. https://doi.org/10.48550/arXiv.2506.16665 (accessed 2025-10-15)..

[11]Flexcompute Inc., “Tidy3D: hardware-accelerated electromagnetic solver for fast simulations at scale,” White Paper (Flexcompute Inc., San Francisco, CA, USA, 2022) available for download.
[12]Flexcompute Inc., *Tidy3D Documentation*, Flexcompute (2024), version 2.2.0.
